# COVID-19 pandemic experiences of students from BAME and White ethnic groups pursuing higher education in the UK: A qualitative comparative exploration

**DOI:** 10.3389/fpsyt.2022.1078479

**Published:** 2023-02-23

**Authors:** Geeta Hitch, Shahaduz Zaman

**Affiliations:** ^1^Faculty of Health Sciences and Wellbeing, University of Sunderland in London, London, United Kingdom; ^2^Professor in Medical Anthropology and Global Health, Brighton and Sussex Medical School, University of Sussex, Brighton, United Kingdom

**Keywords:** COVID-19, pandemic, experiences, BAME, White, students

## Abstract

**Background:**

The new severe acute respiratory syndrome coronavirus 2, SARS-Cov2, more commonly known as COVID-19 continues to be an ongoing cause of one of the most disruptive pandemics worldwide. UK universities comprise students who come from different backgrounds and cultures and the majority returned home or lived alone during the lockdown. This qualitative study aimed to compare the experiences of students from Black, Asian, and Minority Ethnic (BAME) backgrounds and White British students.

**Materials and methods:**

An in-depth interview was conducted with each of 35 students—18 from BAME and 17 from White backgrounds—in their homes on Zoom or Microsoft between November 2020 and May 2021. Interview transcripts were checked for accuracy and were analysed using N-Vivo coding software for themes. The pre-pandemic and pandemic experiences of students were collected in the form of a data, including perceptions of the COVID-19 virus, the physical environment in which students lived, and the effect on their general wellbeing, social interactions, online learning, teaching, and assessment.

**Results:**

Mental health issues were more prevalent among female students compared to males and this was more evident amongst BAME females who had returned to living at home, possibly due to lack of personal space. There was a reduction in personal hygiene in White females. There was an increase in the use of social media, particularly by BAME females. Students mostly disliked online learning and exams. Concerns were also raised about how universities communicated important information to the students.

**Conclusion:**

Based on the recommendations of the WHO in relation to COVID-19, universities need to effectively manage the information they provide to students and build trust through risk communication and community engagement. One important area in this regard is addressing access to mental health services. The reasons why BAME females experience more mental health issues should be explored further.

## Introduction

The new severe acute respiratory syndrome coronavirus 2 (SARS-Cov2) more commonly known as “COVID-19,” emanated from Wuhan, China in late 2019 (1), and continues to be an ongoing cause of one of the most disruptive pandemics worldwide (2). This disease outbreak was declared a Public Health Emergency of International Concern on 30 January 2020, by the WHO on recommendations from the Emergency Committee. To date, this has caused over 6.5 million deaths globally (3). The first official lockdown was imposed on 23 March 2020 in the United Kingdom (UK), when people were advised by the government to leave their homes only to buy food, exercise one time a day, or work for those who could not work remotely (4). The government imposed travel restrictions along with the closure of many non-essential businesses, schools, colleges, universities, and venues such as gymnasiums, theatres, cinemas, bars, and restaurants in order to contain the transmission of COVID-19 (5, 6).

In the UK alone, over 24 million confirmed cases and over 196,000 deaths have been observed to date (4). Due to the lack of approved pharmacological interventions or a vaccine for the treatment of COVID-19 during the early part of the pandemic, measures such as social distancing, the wearing of personal protection equipment (PPE), the use of face masks and hand sanitising gels, quarantine, and self-isolation of vulnerable populations were the mainstay of preventing virus transmission (6). As little was known about this virus in the early stages of the pandemic, there was a good deal of uncertainty about how the spread of the virus was best managed both at a national and global level (7). As there were signs of the number of infections declining after the initial lockdown period, many businesses, and venues were allowed to re-open (7). Many Higher Education institutions (HEIs) across the UK also opened, having been provided with guidance from the government in readiness for the new academic year, and many students returned to life on campus (8, 9). However, as the numbers of infections rose, further local, and national lockdowns were imposed in the UK (10, 11).

The government introduced many restrictions to reduce the transmission of the virus. These included the “Four-tier coronavirus alert levels” (12) namely Tier 1 (Medium alert), Tier 2 (High alert), Tier 3 (Very high alert), and Tier 4 (Stay at home alert) which was introduced following concerns due to the new alpha variant of the coronavirus in Kent and the rising number of cases (13). Tier 1 was a medium-level alert rule which meant one could do all the following: meet with others from different households in groups of six people; there were no restrictions on travel but the social distancing from anyone not in your household or support bubble and wearing of a face mask was advised alongside with no restrictions on the use of transport. The rule of six was applied to gatherings at churches. Sporting events could take place subject to a minimum (1,000) and maximum (4,000) capacity as applicable to both indoor and outdoor venues, respectively. Only 15 people could attend wedding ceremonies and up to 30 people could attend someone’s funeral and up to 15 people were allowed to attend someone’s wake; care home visits were allowed only if specific associated rules were observed such as testing negatively to the coronavirus; observing indoor/outdoor visit guidance and limited to two visitors whilst maintaining a safe distance and wearing Personal Protective Equipment (PPE). Each care home had its own guidance to be followed in addition to these rules. Immunocompromised people were advised to isolate and limit contact with others. Finally, each household was allowed to form a “Christmas bubble” with up to two other households for Christmas day to socialize on Christmas day. In Tier 2, journeys were allowed to work, shop, and hospital but one had to wear a face mask; travel to Tier 3 areas was restricted unless working or deemed necessary. Tier 4 level required people to stay at home as much as possible, leaving their homes only for work, exercise, shopping, or hospital. People were advised to go away with people from their household only or support bubble when travelling with no restrictions on overnight stays. A person could only meet up with one other person in public outdoor places and up to two people could care for someone with a disability or a child. Only essential travel was advised with face covering. Travel between different tiers (since the country was allocated a tier status of 1, 2, 3, or 4) required people to observe the rules of that tier (12).

People could not stay elsewhere overnight unless deemed essential. Unlike restrictions in other Tiers, where all shops along with personal care facilities, hair salons, gyms, and pools could open for a limited time, but hospitality sector was advised to close at 11 p.m. Tiers 2–4 rules had stricter variations on various aspects of the rules according to the level of alertness ranging from restrictions on what people were allowed to do in terms of travel, socialize, go into work and exercise amongst other things. However, around all four tiers, rules were often confusing for the public and this meant many were not followed (12). Additionally, many people who could not work from home were required to go to work (14). Such interventions also had the potential of introducing inequalities for those in low socioeconomic groups and in occupations that require going to work (14).

Many students were forced to return home and study online in isolation from their friends and campus life (15). Traditional exams, lectures, and placements were halted, and academics were required to develop online curricula that were novel and engaging (16). This had the potential to lead to inequality, particularly among international students who were dependent upon the availability of technologies and access to the internet (17).

This study was initiated in October 2020, and at that time, there was a dearth of literature on the impact of COVID-19 on UK students. One area not investigated at all was whether students from BAME and White backgrounds were affected differently by the pandemic; hence this was the primary aim of this qualitative study. The objectives were,

1. To explore the overall experiences of students regarding the social, psychological, economic, and educational aspects of their lives prior to and during the pandemic.
2. To identify what, if any, differences in those experiences existed between BAME and White students in UK universities between November 2020 and May 2021.

## Materials and methods

### Study design and setting

A qualitative approach was used for this study to explore the students’ experiences of the pandemic as the UK was under a strict stay-at-home order (17) from 23 March 2020 to 12 April 2021, because of the COVID-19 pandemic. Therefore, the study setting was the researcher’s and the interviewee’s home environments within the UK or abroad. A qualitative study design using semi-structured interviews (18) was selected since the aim was to explore the thoughts and feelings of individuals during the pandemic period.

The interpretive ontological and epistemological approaches assumed that reality was a social construct and that the reality depended on the mental structure and activity of participants, based on their own construction or interpretation of reality (19). Thus, the research question was answered by gathering participants’ beliefs, understanding, and experience as they described and explained events, leading to the collection of contextually rich data. A non-probabilistic purposive sampling approach was used to recruit potential participants from various universities (20). To increase the sample size, a snowball sampling technique was also used, whereby participants were asked to inform their friends if they were interested in being interviewed (21). One-on-one qualitative semi-structured interviews were conducted online using video conferencing on Zoom© or Microsoft Teams© platforms, once a pilot study was conducted on four participants to ask for their feedback on the relevancy of the questions and overall content of the interview guide developed to aid the process (22). It consisted of the following themes adapted from Austin and Gregory (23), with the breakdown of the focus of the prompts in brackets:

1. Demographics.
2. Pre-COVID-19 life (social, economic, and general wellbeing).
3. Life during lockdown (social, economic, and general wellbeing).
4. Post-COVID-19 expectations from life (social, economic, and general wellbeing).

Each domain had structured questions and prompts to guide the participant. The interview guide was followed for all the respondents and, as new information emerged, these questions were added to the guide. All respondents were asked identical questions but not necessarily in the same sequence. The reason was that once the respondents started talking, they might provide answers to questions that were to be asked later in the interview, thus, there was no need to ask them for the information again by sticking to the same order of question-asking for each participant. New questions emerged during the course of the interviews and as the interviews evolved in themselves. All the elements of the question protocol were covered throughout the course of the interview.

Prior to the interview, participants were informed about setting a time and a quiet place for privacy to engage in the interview. Fluency in English was not a prerequisite as it was assumed all individuals had the required level of English to gain entry into a UK university. There were no incentives in the form of payments or similar. Each interview lasted between 25 min to just over an hour, with an average of 45 min per interview. The information that emerged from the interviews was subjected to inductive analysis into themes and subthemes (24).

Data were collected by GH between November 2020 (during the time of the second lockdown) and May 2021 (just after the final lockdown when restrictions were lifted). Each participant was informed about the process prior to the interview both *via* email and before the interview took place. Their verbal consent was recorded. All participants were informed they were free to withdraw from the interview anytime they wanted, and or did not have to answer any questions or divulge any information if they felt uncomfortable. Each interview was recorded, and the transcription facility was used to produce a verbatim account. Participants were anonymized by coding them as follows: B for BAME and W for White ethnic background respectively; M and F for male and female, respectively, and a number were assigned to each participant and GF1 for the gender-fluid person. As qualitative research focused on the meaning and understanding of the research topic in question, it had its own high internal validity. All transcripts were reviewed by SZ for reliability. All transcripts were sent to each participant for feedback, for transparency and accuracy, and validity and reliability (dependability and credibility) (25) of the data gathered. Throughout the discussion, verbatim quotes were used to support relevant themes. Recordings of the interviews were sent by GH to co-author SZ to ensure validity and reliability. Multiple data sources of research findings are used to converge information in qualitative studies to increase the validity (accuracy) and credibility (trustworthiness) of the study by the process of triangulation. Triangulation was not possible in this study as only one method and one population sample type of participants were used (26).

### Ethics and participant confidentiality

The study was granted Ethical and Research Governance Approval by The Brighton and Sussex Medical School Research Governance and Ethics Committee (RGEC); Reference number ER/GH216/4. Informed recorded verbal consent was obtained from each participant.

## Results

A total of 35 interviews were conducted with students from 2 to 4 years of their undergraduate studies (30) or enrolled in postgraduate programmes (5); the majority of these were studying medicine, dentistry, pharmacy, or law. The cohort had an average age of 21.7 years and comprised 20 females, 14 males, and one person who self-identified as gender fluid. The cohort was further divided into either BAME or White groups. Eighteen were of BAME ethnicity and 17 were of White ethnicity. Of the 18 BAME participants, 12 were female and six were male, while in the White ethnicity, eight were female and six were male and only one was a gender-fluid person. The former included 18 students of Indian, Bangladeshi, Arab, Chinese, Mixed race African American, Persian, Afghan, and Nepalese backgrounds, while from the latter, 17 students were of British, French, Romanian, German, and Austrian decent.

### Themes identified from the interviews

The overarching categories of students’ experiences were those prior to and during the COVID-19 pandemic. These were further divided into four main themes and further subthemes as shown below:

1. General wellbeing (Initial response to the pandemic; Impact of news about the virus on news and social media; Ethnicity and experiences of racism; General wellbeing),
2. Physical (Living at home and personal space; Personal hygiene; Self-care: Exercise/Diet; Maintaining effective infection control practices for the safety of self, family, and others; Vaccinations),
3. Social (Life at home with family; Relationships with friends; Using social media to connect with the world; Communication from institutions and government; Cultural festivals; Concerns about BAME healthcare workers),
4. Educational (Managing online lectures; exams and disability; Loss of experiential learning and student identity; Return to campus; Graduation-is it happening?).

### General well-being

Everyday student life experience pre-pandemic was the same for all respondents with respect to a normal day. No specific differences were found either between genders or the two ethnic groups.

*BM4: “As a 3rd year did a lot more socializing. Depending on what was on that day, go to university and then come back usually, go out at least once a day somewhere with my flatmates and then we’d spend a lot of time at home. I’d speak to my friends who are in different cities on social media and then go to sleep really”*

*WF1: “One of my friends would pick up another one of my friends and I, and then we’d all drive to the unit, and then we’d go to the café … and then, yeah, it would just be kind of a going in between lectures or workshops and maybe the library sometimes”*

There was no difference between BAME males and females regarding the knowledge of COVID-19, as most students also had a good understanding of how the virus was transmitted and appeared to be fully aware of the risks associated with it. Students appeared to have enjoyed life pre-pandemic.

When participants were asked if they had heard of the coronavirus and how it was transmitted their responses were as follows:

*BF2: “We had studied virology and little bit, and I’d heard about SARS, and I’d heard about sort of obviously the Ebola virus and swine flu and small-scale epidemics, like that and Spanish flu. And but I never particularly focused in on the Corona viruses or anything.”*

*“And so, from my understanding, it’s by droplets, person to person transmission. So, you can kind of catch it on your hands and get droplets on your hand and the spores are in the droplets and then you can touch something and then if they touch their face or anything. It’s kind of transmitted like that”*

*BM4: “yeah because I heard about it in the start of January, but I just, I just saw passing articles I didn’t take it seriously, …yeah and then I see it’s kind of. it’s close to home …”*

*“Get the virus from the population or through free droplets, but it can be through…. contact.”*

*WF1: “Well, I mean, I think most of us probably like in Europe, probably started hearing about it sometime in January: Yeah, but it just was constantly like, oh, it’s just in China thing and then like, oh, it’s just here or there, like, it won’t be here. And then once a few cases did start popping up in Europe, we were all like, oh, it won’t be that bad…. transmitted through the air. I should really know much more than that”*

*WM5: “Before the lockdown I’ve heard about it in the news”*

*“As far as I know, as far as I know, it’s airborne but I’m not fully researched on it”*

### Experiences of the announcement of the first lockdown and living at home

Many of the students reported feelings of excitement when the first lockdown was announced. Terms such as “a scary excitement,” “surreal,” “dystopian,” and “bad Sci-Fi movie” were not uncommon. There was no difference in the experiences in either ethnic group or between the genders. For example:

*WF4: “It was a moment of scared excitement, I’d say, I’m thinking. Oh no is so dystopian I remember thinking, this only happens in the books I used to read when I was a teenager Everything this is completely dystopian You know, borders are closing and country there’s no traveling and I mean it was…. It was a moment of scared excitements”*

*BF2: “So I don’t know if you’ve watched the show- Black Mirror? And it kind of feels a little bit like that. So, kind of like quite Sci-fi and It’s something that you’d kind of read a book on and be like, oh wow that’s so strange. Like, how many people live like that, but it’s like, no, because we’re living like this and it’s just strange honestly like it feels surreal”*

*WM5: “The excitement was sort of the adrenaline rush you get from this as a new situation, a new environment, there’s a pandemic, things are different, this is kind of turning into like a bad Sci-fi movie. But then, then things changed as we started realized…. oh, dear things are changing….”*

*WF6: “I actually thought it was kind of exciting right, so we were all working from home. And that’s the new era. And what’s happening now and had never experienced that before”*

*BM1: “…this big hullabaloo trying to get toilet paper and all that stuff. Which is kind of exciting, but a bit scary. And then, um, it was difficult because all this was happening in the UK and none of it was happening yet in my home country”*

Many students enjoyed seeing the family and living at home for the first fortnight. Things changed rapidly for many, especially for the BAME females, who were expected to fit into the home environment while living at home. As one student said:

*BF2: “They were happy to see me yeah, I haven’t been home properly in 3 years, but of course there were difficult times. I didn’t have any personal space at home. I share my bedroom with my brother who has mental health issues but me moving back changes the dynamic different sleeping patterns he’s a guy I’m a girl, we share a room is difficult, but it was difficult in terms of coping with his mental health issues and his lashes (when lashing out)”*

By comparison, the White students who lived at home, all had good relationships with their families and were not expected by their parents to fall into the household routine, unlike the BAME students.

*WF5: “you can sit around, you don’t have to go anywhere, do anything”*

Black, Asian, and minority ethnic (BAME) students felt pressured in moving back home to their homes by their families and BAME females, in particular, felt anxious about moving back home, not wanting to do so.

*BF4: “because my family situation is a bit difficult in the house and mum was–“now I have to spend even more time with people I don’t want to be spending time with””*

There was a strong sense of self-reported despair amongst the BAME students who had returned home as they clearly felt “suffocated” at home especially when the second and subsequent lockdowns were imposed.

*BF3: “I feel this lockdown was definitely more impactful on my mental health than the first one. I started feeling very suffocated…. genuinely have been in tears and…when you’re at university, you’re in your own space so you decide when you are going to do whatever you want. Whereas, in a home when you’re with your parents and family that’s more around everyone’s routine. So, it kind of stops to you as a student from kind of having your own. That’s frustrating,”*

*“…. There’s just that disparity between the two environments of living with your friends and your family. Before exams if I’m very stressed at unit, I can be up to four or five (in the morning). But I just feel my parents don’t; they just think that that’s strange or I am putting too much stress on myself, or their understanding of my education is different to the way I deal with them. Right now, I don’t really feel like a student. I just feel like I’m doing my A levels. Again, I don’t feel like I’m a university.”*

A sense of self-reported perceived anxiety was also heightened in BAME males whereby those who did not share a room stayed in their rooms only coming out to eat as affirmed by this student:

*BM3: “The thing is I’m just in my room. I basically come out of my room to have food and then go back into watch YouTube or something I’ll go to watch the lectures or something like that”*

In contrast, most of the White students appeared to want to be back home:

*WF5: “I need to get home. As soon as possible”*

Where students were co-habiting with their partners, the BAME male felt anxious, whereas the White male was more welcoming of his partner, as this affirms:

*BM4: “at the start it was strange because I just felt you didn’t have a break from them, but then you have got used to it”*

*WM5: “We got to spend more time we got to spend a lot of time together that we may not have been able to spend”*

Black, Asian, and minority ethnic (BAME) females appeared to have suffered more from feelings of anxiety, sadness, loneliness, and depression during the pandemic. All the females who received counselling during the pandemic went back home to live with their parents. There appeared to be a clear association between mental health issues and lack of autonomy, personal space, and loss of student identity in these individuals. Anxiety resulted from not having the space and the appropriate privacy to do online learning and lack of personal contact with their university friends. Feelings of frustration were expressed by BAME females about parents not having that understanding of how they studied or when they went to sleep or woke up. They were expected to conform to a life they had left behind. It was evident that a lack of personal space had the greatest impact on BAME females, the majority of whom (9 of 12) were from lower socio-economic backgrounds and had returned home to live with their families during the lockdown. For example:

*BF2: “It’s awful. I don’t have a desk in my parents’ house. I’d have to sit and work on the dining table. But then my sister and my dad are taking business calls. My dog is starting to bark”*

*BF12: “Finding the time to revise and finding your own space in the house is quite difficult because I share bedroom, and to revise is quite difficult. I have three siblings so the six people living in the house so it’s quiet, packed…”*

By contrast, 16 of the 17 White students interviewed had their own private space in which to study. They either had their own desks in their bedrooms or could access their parent’s home office. For example:

*WF5: “I am such a homebody I love being at home. I’m currently in this office in my house, which is in our back garden. So, I just come out here. I sit with my laptop.”*

*WF7: “I had my own space, so I had my room um I could still talk with my friends and everything they (parents) weren’t asking too many questions”*

Some students were worried about the impact on their families. For example:

*WM6: “I was a bit excited … But my dad got furloughed … and he was he was obviously worried that he was going to lose his job which he did in the end. But yeah, it was it was a scary time for my family because they weren’t doing the best financially as well”*

The WHO defines mental health as “a state of well-being in which the individual realizes his or her abilities, can cope with the normal stresses of life, can work productively and fruitfully, and can contribute to his or her community” (27). Of the 35 participants in this study, 65.7% (*n* = 23) reported having some form of mental health issue such as anxiety, depression, eating disorder, and borderline personality disorder. Thirteen were of BAME ethnicity (nine female and four male) and 10 were of White ethnicity (four female and five male). One was a gender-fluid person. Pre-pandemic, four BAME and four White ethnic females and two BAME and two White ethnic males had already been medically diagnosed with depression and/or anxiety, eating disorders, or borderline personality disorders. During the pandemic, these students reported experiencing overwhelming feelings of fear, panic, and anxiety. Some students reported receiving prescribed medication for these mental health issues.

*WF3: “I was diagnosed with borderline personality disorder last year, and I’ve been taking medication, I’ve been going to therapy. I’ve been doing really well until the pandemic started because I had my routine which kept me going. And then my routine was taken away. So, I completely lost my momentum, and everything was just very…. my head was very cluttered.”*

*“I wouldn’t say that I’m depressed because I’ve been depressed and it’s not like that. But I feel myself going there…. I’m on the way to depression”*

*WM2: “(I became a career of parents who are both disabled). It’s overwhelming. I’m currently on medication for stuff and yeah, so….”*

*BF2: “And I do have, I have been diagnosed with depression and anxiety I currently take medication for that…”*

Counselling (28) or “talking treatment” involves a mental health counsellor and a client working together to seek out cognitive, behavioural, and emotional aspects of mental health problems and for the client to then acquire relevant strategies to overcome these challenges in order to live with good mental health (29). There are different types of counselling treatments dependent upon the client’s mental health problem. Counselling can be sought for many of life’s stresses as well as specific mental health illness (28, 29). Six BAME participants reported receiving mental health counselling pre-pandemic, compared to three students of White ethnicity. Another eight students reported receiving counselling during the pandemic of which 75% (*n* = 6) were of BAME ethnicity, five of these (83.3%) were females. In comparison, only one white male and one female reported the same.

*WF8: “stressed worried…. lot of anxiety… then I did access some counseling services”*

*BF12: “I get really, really stressed out so stressed out… I actually went for …like a block session of counseling”*

*BF2: “… and I’ve learned quite a lot of ways to sort of support myself throughout the past few years. You know whether that’s sort of taking a step back from everything or going to chat to someone about how I’m feeling and having a bit more of a deep conversation about it.”*

*BF11: “It was horrible but yeah and after it was just mixed feelings… so my feelings were just going like from super happy to super exhausted …. counselling”*

*BF4: “I overthink and stress and panic a lot like my mental health hasn’t been too great I had an excellent supervisor (a counsellor also) who helped all the time”*

Overall, there was a general pattern of mental health issues being more prevalent amongst females, particularly those from BAME ethnic backgrounds during the pandemic. There were also three cases of mental health issues recurring in students of White ethnicity who had a prior history of this condition.

### Changes to personal hygiene

The UK government provided guidance on the general principles of cleaning and hygiene for the public. This included information on the mode of transmission of the virus from aerosols and droplets coughed or sneezed by those who were infected and passed onto others who came into close contact with them. The public was advised to keep their distance from others, wash their hands regularly, avoid face touching, and clean surfaces in order to curb the transmission of the virus (30).

In this study, students were asked if their personal hygiene routine had remained the same or changed as a result of the pandemic. Fifteen reported no substantive change during the pandemic; the majority (10) of whom were BAME. On the contrary, 11 students admitted that their personal hygiene deteriorated markedly, 7 of these were White females. Nine students developed an obsession with frequent hand washing during the pandemic. Of these, eight were female, of which six were BAME. It was a general observation that in households where students had their own room and personal space, the importance of routine and personal hygiene were not a priority. Interestingly, hand washing frequency increased in 13 students who were or had been on medication and/or receiving counselling for mental health issues. Six of these were BAME females; one was White female and one was gender fluid. The reason for the increase in hand washing is unclear but may have been associated with either close adherence to the guidance in order to keep themselves and their families safe and /or was enhanced in those who were already predisposed to some form of mental health issue.

*WF1: “I always wash my hands because they feel gross. So now I washed them longer”*

*WGF1: “during the first look down it (my hygiene) went completely out the window, because I was in such a state that I was too scared to go into the bathroom”BF12: “So I have I’m a bit of a like I’m very sensitive when it comes to hygiene like very, very sensitive and like to the point where like I don’t tell people how sensitive I am “cos I’m paranoid””*

*“I’ve changed the hand wash in my bathroom from normal hand wash to antibacterial hand wash and I checked I washed my hands, but I’ve always been in the habit, even before corona time when I’m in like when I’m in the bathroom I’m always washing my hands, like every 5 s …. I have this habit.”*

*WF3: “I have no motivation even to brush my teeth…. which is really embarrassing because I’ve been up since 10 (am) and I haven’t done anything but lay. I moved from the bed to the chair.”*

*WM5: “it [my hygiene] was better before the pandemic started, now not always on the top of my priority”*

*WF8: “it [my hygiene] completely fell apart…. I was showering maybe once every 2 weeks, because everything stopped”*

### Issues related specifically to BAME students

BM6 was the only Chinese–Malaysian student in the cohort. His account of racial abuse left him feeling anxious and frightened. Extracts of this account are given below:

*BM6: “I’ve had teenagers literally throw sticks at me… it was quite a terrifying feeling to feel that it’s just horrendous.” I’ve definitely had Anti-Asian racism levelled against me …. close to being physically abused… just really scary, I was numb.”*

*“That has really caused me to be more socially isolated. I just don’t to go out that much anymore really and I feel that’s a lot of that’s a sentiment like a lot of Asian people feel especially regarding this issue”*

Celebrations of Christmas, Diwali, and Eid were all affected by the pandemic as lockdowns came into effect. In the UK, the newspapers were reporting spikes of cases in the North and in areas of higher deprivation and poverty. The areas included were places like Leicester and Manchester where there is a high concentration of ethnic minorities, especially south Asians. Students were concerned about not being allowed home for their national festivals and questioned why this was not applied to Christmas as well. For example:

*BF2: “I think that the government is currently focusing on White wealthy individuals, rather than someone like me who comes from working class background, who is a person of colour, who does have people from a different heritage; they’ve lifted it for Christmas and then just didn’t really make it for that festival”*

### Routine, diet, and exercise

Some students made an attempt to organise a routine into their daily life but found this to be boring since they could not go out. However, this was more pronounced amongst BAME females who were living at home and amongst those who had jobs during the pandemic.

White female students were the least likely to exercise. For the BAME males, also experienced a lot more self-reported anxiety and their eating habits changed since for the majority, eating decreased and smoking increased. For example:

*BM4: “Anxiety increased mainly due to the changes in my routine; smoking increased; food decreased”*

*WF3: “I spend hours on Tik-Tok and on Instagram. I think I could spend that time maybe exercising because I gained 15 kilos, since we started lockdown. I started eating for comfort”*

However, the majority of the students went walking once it was allowed as part of their daily routine.

### Fear associated with public spaces

Nearly half of the students expressed concerns about travelling on public transport, largely because of the public’s failure to wear face masks or maintain social distancing. Many students felt that regulations were not enforced rigorously enough. For example:

*BM10: “I think before I wasn’t as reluctant to use public spaces, as I am now”*

*WF8: “I especially don’t like a full bus and people aren’t wearing their masks”*

The majority of the BAME females stated they avoided public transport altogether. White students were fine using public transport as long as they took preventative measures. However, most of the White students, and in particular, females, had use of car to travel and so were not overly concerned.

All students reported using face masks, hand sanitisers and maintaining social distancing wherever they could. Although there were no differences in the behaviours of either ethnic group or between genders, half of the BAME students expressed concerns over transmitting the virus to their parents or grandparents. It is noteworthy that all of these students were reading professional healthcare degrees and therefore had a heightened awareness of the risk factors associated with the transmission of this virus.

*BF10: “I was very scared I took time off work; my mother was a high-risk individual”*

*BF8: “I try and minimize going out. because I live with high-risk people”*

### Vaccinations

There were some interesting findings concerning vaccinations. Over a third of BAME students said that either they or their families were reluctant to take the vaccine because it had only been available for a short time and because of reports of side effects. For example:

*BF10: “I don’t feel comfortable taking the vaccine …lots of side effects. Risk of blood clots…different types of vaccines …you don’t really know”*

Some BAME families preferred to take traditional remedies and not the vaccine, as was highlighted by a couple of students:

*BM4: “my mum believes that, if she if she has manuka honey, or if she has herbal teas and things like that it will boost your immune system”*

*BF8: “the older uncles and follow these Indian traditions of drinking turmeric and milk or some stuff like that, and then the uncles and aunts believe they are not going to get COVID”*

By contrast, the majority (13 of 16) of White students stated that they and their families would take the vaccine.

*WF4: “I do think I’ve been fortunate enough to have a family that’s quite educated and three or four of my family members are actually in medicine. My grandfather is a doctor and he’s received it, my grandma’s waiting to receive”*

### Life during the pandemic

#### Social media and connection with society

TikTok©, Instagram©, Snapchat©, and Twitter© were the most popular social media platforms used. Many students also joined Linked-In© and accessed Twitter© for instant news updates. Overall, the time spent on social media doubled in all groups during the pandemic, some spent as much as 10–12 h a day on social media. The average use of social media was higher in BAME females (6.8 h) and males (5.8 h) compared to the White ethnic group (4.7 and 4.3 h for females and males, respectively). The likely reason why this use is higher in BAME students is that they are living within the close confines of family and do not have personal space, thus, communicating with friends on social media may be seen as a form of escape. Interestingly, many students reported that they constantly monitored news feeds about the pandemic during its early stages but then found it distressing as the situation progressed. For example:

*WM3: “so depressed at how deadly everything was”*

*BF4: “It’s kind of constant exposure. Yeah, when I’m kind of feeling down. I try not to go on social media, it’s just, it’s just there all the time”*

#### Communication and learning support

Nearly all the students expressed anger, frustration, and disappointment at the lack of and sometimes the level of information communicated to them by their HEIs and the Government. About a quarter of students believed not enough precautions were being taken and many commented on their confusion about the various changes in guidance given by the government. Overall, the answers provided by both ethnic groups and genders were very similar and largely reflected those of the general public. There were no differences between either ethnic groups or genders. It was evident that many found that communications with their host institution were overwhelming and not particularly informative. Some also had problems obtaining relevant advice concerning their learning support needs. Some felt the information from the HEIs was not even worth looking at as it told them nothing new as was affirmed by a few students:

*WF2: “There have been a lot of updates emails and the problem is that they often aren’t updates so every week, it’s just basically an email to say will let you know if there’s any information, but it’s posed as if it’s going to be”*

“*BM1: Getting all these emails (from the University) telling you there’s this coming and this is coming. We’ll keep you informed. Something’s happening in the background, but you don’t have to email me every day. Tell me if something’s happening in the background.” When I asked: “Oh, I have a question, because I don’t know a little while back you said, you know, if you’re struggling with exams or this or that, email your academic advisor. The university responded: “Sorry, ask your academic advisor.” And I emailed my academic advisor, and he was like, well, if you refer to the email [the original email sent by the University], they tell you a bit about it in there. So, I looked at that email. Again, there’s nothing in there about it just says, “will inform you later.” So, it’s like it just gets passed along and instead of passing me along, maybe just tell me when something concrete happens”*

*BF4: “It’s like there’s nothing left and never any information on what’s happening with the university … and it just feels like we’re left to fend for ourselves”*

*BF10: “I felt like they were providing support to providing general advice that was already being relayed on the news. And that Boris Johnson would give information and then [the University] was just really giving that information back to us given”.*

*BF9: “Okay, this is what you need to know (from the lecturers) and ABC the end that’s it! And whenever we asked questions, some of them were very dismissive actually that we don’t have time, maybe at the end; It’s ridiculous now that I don’t feel like we got the teaching or the resources or anything”*

*WM4 (student with dyslexia): “pre-covid we would go into a room where it’s usually me and maybe one other person and there’s someone there to read us the question. Now (during covid) obviously, it’s on online, and the exams are quite stressful sometimes because you have to scan in all the work within the time limit, whereas pre-covid, you usually just hand in the paper and now if I don’t understand a question, there’s nobody I can really ask about it, because the lecturer may not get my email in time and you don’t want to be sitting twiddling your thumbs waiting and sometimes it is just a matter of trying to understand what the question is asking in simple terms, and there’s nobody there to read it out. So, um, it’s definitely a lot harder than usual.”*

*WF3: “I personally messaged the Disability Support because I needed help, and the Disability Support took about almost 2 weeks to answer”*

*WM4: “In fact, I’ve had the needs assessment, but it’s taken us well about 2 years to get to this point.”*

Whilst a few students mentioned receiving support from their academic advisors, this was not always the case. Many commented that the university had not put much thought into the quality of the lectures since old, pre-recorded lectures were often posted for students, and technical support for students with learning disabilities was not forthcoming and this left many frustrated. Many also felt they were paying far too much money for an “Open University” degree. For example:

*BF4: “And I’d say the only way they adapted is move everything online, but it’s not really value for money to sit in my own room on my laptop”*

*WGF1: “I think they [the University] moved the times of their assessments back, which was helpful, but I just think it wasn’t enough. You kind of felt like the university didn’t really know what to do, which is fair. I don’t feel very supported by what they [the University] did do.”*

#### Online learning

Online learning, teaching and assessment has to be organised by the academics in order to engage and enhance students’ learning. Students self-reported experiences of online learning were rather mixed in terms of content, quality and the general organisation of online delivery as highlighted by some students:

*BF3: “It’s transformed to being behind a screen more, so things like FaceTime, Zoom and Skype, etc., staring at a screen for x hours of the day”*

*BM4: “Pre-covid, if you didn’t understand something it was easier to ask questions. With online lectures you can put your hand up if you want to ask question and with Zoom calls it’s really easy to talk over people accidentally so you’re constantly stopping and starting you can never get into a flow of conversation it just doesn’t feel natural. I liked them. I don’t really see a big change. I’ve actually had an improvement in my grades because my attendance wasn’t the best basically, but online lectures are easier to attend so my grades have actually gone up. However, I’m still losing out on my workshops where I would have had patient contact, and you can learn faster in my opinion in person”*

*BF3: “We’re doing our masters, and some [students] started like their lab work for their dissertations and they’re still expected to submit a dissertation, but then have to stop their lab work. So, I think they’re really worried and confused. And at that point, it was kind of hard to get in contact with some lecturers don’t respond as quickly. So, I think it’s sent like a panic through them and what would have, what would happen in regard to their exams”*

*WF1: “Anyways, um, so yeah, …he [The lecturer] kind of just put up the stuff that had been recorded the year before, and the sound wasn’t good. And it was kind of difficult”*

*BF2 (a medical student): “We have problem-based learning, (PBL) small group work to solve case studies. You always hear stories from people in years above, about how close they all became as a PBL group and how they had cake rotas and secret Santa. But obviously, because we’re doing it online, we don’t get any of that. There are people in my PBL group I don’t really know what to say to them, because I’ve only ever met them at screen.”*

*BF4: “Exams: it doesn’t feel like a real exam. It just feels like a long essay”*

*BM3: “The exam experience I may think, to be fair, it’s not all bad because there’s a lot of freedom to what we can do. But I would say, I could have revised more compared to if it’s online and its open book I would revise a bit more. I would have been taking it a bit more serious, but that’s the thing- it’s all diluting the seriousness of the exam. Exam is a period to be like stressed and like you know to be taken seriously, but now it’s just it’s a bit more “oh yeah I can actually you know calm down a bit just not take this as serious” as I should have done [if it was in an exam hall]* and to be fair *you don’t have to actually stress a lot”*

*WM2: “exams were obviously a lot easier because they are now open book so when I was like revising most of the day, I found myself like stopping and thinking going, “Why am I revising it’s an open book exam?””*

*WM6: “I think even the lecturers didn’t really know what was going on, to be fair, so I don’t blame them for not getting information and even quitting, but I think the information getting out was a bit slow, especially in regards like exams and stuff coming, I think we all just didn’t really know”*

*BF8: “It was hard to be on Zoom like I couldn’t answer questions as much; I didn’t want my microphone on, because mum’s on the phone. Indian parents have really loud voices when on the phone; my sister is trying to do her college lectures; the dog is barking and I live above the shops, I can hear customers coming in. There is no personal space in Indian homes”*

*BF8: “In terms of experiential learning, we had virtual placements which resulted in us having a lot of problems with our computers in running the software.”*

*WF4: “And it [the University] really struggled to set up online learning. I remember going to a class and having had none of the content because the teacher couldn’t figure it out. And I remember sitting in front of my laptop and then Zoom just didn’t work. There was nothing that was working. I was just like. This is more anxiety that I can deal with…. It was just too much. So, they [the University] were like, if you feel like this is too much, please terminate the year and you will not be held accountable, you will pass”*

*WM5: “regarding online etiquette, there were always problems - a computer not working, Students not showing up, leaving microphones on so you hear their conversations in the background, not hear what the lecturer s saying, or engage meaningfully in a class discussion, online lectures can over run a bit more”*

#### Financial burden

Over half of the participants interviewed undertook part-time work prior to the pandemic, but only a minority remained in employment during it. Of the 35 participants, 7 out of 17 white participants were reading professional healthcare degrees such as pharmacy and therefore had plenty of opportunities for part-time work. Five out of seven of these received bursaries from their universities as first-generation scholars. Of the BAME participants, 15 out of 18 were reading for professional healthcare degrees such as medicine, dentistry, law, and pharmacy, and worked on a part time basis. Of these 18 participants, 10 either received bursaries from their universities as first-generation scholars or were on maintenance loans. Whilst this did not form part of curricular placements, it reflected upon the relationship between the education program that students were following and the part-time jobs they kept during COVID-19. It was noticeable that White females from higher income families did not need to work and were financed by their families. The average monthly income for students from lower-income families, who also received bursaries of £500 per semester, was around £750 per month and for the less disadvantaged students, it was between £1,000 and £1,500 per month. It was also noticeable that the majority of BAME students worked in healthcare and so were able to keep their jobs. Many complained that both the student accommodation and tuition fees should have been reimbursed and students offered more financial help. For example:

*BF4: “I technically did not have an income. The money I got from student finance that I had saved was the only money I had. Also, had the First-Generation Scholar Bursary that the university offered (£500 per semester) and received student finance. Spending on rent increased as I had moved to a different house and the landlord had put the price up of the property, so it got more expensive”*

*BF12: “Prior to pandemic I only worked 5 h a week, however during the first wave of covid I worked a lot more hours hence were earning more money (approx. £500/600 per month). The only other source of income I had was the first-generation bursary from the university (£500 per term) … so I’ve taken out a what’s called a tuition fee loan for every year, (4 years); I have been repaying in chunks”*

*WM2: “they’re almost demanding £9,250 whereas it’s all online where you’re not getting your money’s worth”WF1: “My mum - She would send at least £1,400-£1,500 every month”*

*BF10: “in terms of financial support for those who don’t live in a university managed accommodation, the least they [the University] could have done was provide a guidance on how to approach our landlords.” “I wasn’t getting furlough and I needed universal credit they [the government] said there’s nothing that they can do so, I was in a bit of a financial struggle in terms of money, so I increased my credit card limits”*

*WF5: “I don’t think we should be paying the same tuition fees. The online learning, they [the University] wanted to maybe take guidance from the Open University charges or another online university”*

### Concerns about BAME healthcare workers

Many BAME students expressed concerns about the number of COVID-19-related deaths among BAME healthcare workers. For example,

*BF2: “the only other comment I’d have to make is how bad it is [for BAME healthcare workers] in the NHS to work and how underappreciated they are, and the fact that BAME doctors and nurses were, in terms of mortality the most affected. And I think that was very, very upsetting for me as a person of colour”*

*BM3: “I mean my mum’s a nurse so she I think she had enough of it because it’s always so just very busy in the ward right now and there’s a lot of ill people - and she’s just gotten tired of it, and she also had COVID”*

### Student identity, return to campus, and graduation

The loss of student identity was evident amongst all participants and is exemplified by the following:

*WF6: “Well, now I feel like just like a person with a laptop, to be honest”*

A majority of the participants stated that they found it difficult to remain motivated to study from home and expressed a sense of frustration about the whole situation. This was particularly evident amongst BAME students.

*BF11: “now for being just at home you’re not even allowed to go to a library to a cafe to study anything it just drops my motivation’s so low and also because of the lectures are online yeah you don’t feel you are really studying”*

On returning to campus, majority of the students felt it was safe to return but with mixed feelings:

*WF2: “when I did go in, it was, it was very eerie, very strange”*

*BM1: “I absolutely hated it. Because when I went on to campus, it was just so it wasn’t the campus I knew if that makes sense, but it just there was no people so sterile feeling”*

On graduation some felt there was no formal closure on this student life for them:

*BF6: “you have 3 years of frustration and everything, your graduation is snatched off me”*

For others it was more about future prospects:

*WF3: “So who’s going to hire people now when those are already in place are firing their current staff”*

## Discussion

Many differences were identified in the experiences of the COVID-19 pandemic between White and BAME students and between the genders in this study.

### Personal hygiene, diet, and exercise

Reduced personal hygiene was more evident amongst a proportion of White females. They felt there they had no structure or routine to their days and because they did not share their personal space with other family members, they could stay in their pajamas and “jump” from their bed to the desk if and when attending online lectures. A study assessing personal hygiene amongst Bangladeshi university students found that over half showered daily and only a third changed their clothes on a regular basis (31). In this study, reduced personal hygiene was more evident amongst a proportion of White females. In this study, the majority of males from both ethnicities washed and maintained some form of routine in their daily lives. A minority of students from both ethnic groups and gender had part time jobs in local pharmacies or hospitals which they maintained during the pandemic, and this provided a routine and a reason for them to maintain their personal hygiene.

The majority of students spoke about being bored at home, with nothing much to do, and each day is the same. This was similar to findings in other studies (25, 32, 33). Many started cooking whilst at home during the first lockdown as part of participating in domestic chores. A very small minority indicated their smoking habits, food, and alcohol consumption increased; however, the majority did not smoke and said their alcohol consumption had decreased whilst living at home. The findings of one study amongst Bangladeshi students showed an increase in smoking habit (31). The majority of the students in our study took the opportunity of taking an hour’s walk once the restrictions allowed. The Hunt COHORT study has shown that whilst a small amount of exercise prevented the future onset of depression, it did not alleviate anxiety (34). In this study, only a very small minority felt anxious to go out. These had a pre-existing diagnosis of mental health illness and included female students of both ethnicities. A recent study examining the psychological impact of COVID-19 and the quality of life in women in Hong Kong showed that they experienced considerable levels of depression, stress, and anxiety as the pandemic progressed possibly due to a lower quality of life as they were expected to take up household responsibilities (35). A systematic review has shown that the risk factors for COVID-19-induced adverse psychological responses included a younger age group of whose age was less than 40 years, female gender, a low socioeconomic status, and low educational levels, all of which were associated with many of the BAME first generation diaspora students in our study (36).

### Physical dimensions

The “Build Back Fairer: The COVID-19 Marmot Review” (37), highlights that the high and unequal death toll due to COVID-19 was contributed to by inequalities in social and economic conditions before the pandemic. Certainly, this study identified many BAME students from low socioeconomic backgrounds who returned home to live with their extended families and in households where there was a lack of space making isolating impossible. They became infected with the virus which they subsequently transmitted to their parents due to their living conditions. In comparison, not a single White student in our study developed COVID-19.

The majority of the BAME families in our study were essential, frontline workers which effectively puts them at a higher risk of contracting the virus (37–39). Some students also mentioned co-morbidities such as diabetes and hypertension and asthma within their families. Research has shown that COVID-19 is prevalent in BAME populations which have a higher incidence of such diseases. another study highlighted associations between socio- demographics, ethnicity, and COVID-19 infection risk in the UK (40, 41). It was noteworthy that whilst COVID-19 rates in BAME communities living in deprived areas were higher in England, this varied within sub-groups of BAME ethnicity (40–42). For example, Asian/Asian British had twice the risk of COVID-19 compared to White participants and the risk was as much as threefold higher for Chinese and Black/Black British participants. They also point out the “dose-response” effect of the greater the deprivation, the higher the risk of contracting COVID-19. The authors also noted that there was an association between cardiovascular conditions and non-communicable diseases such as obesity (40). Similar observations were found in a study in the US (43) the authors point out that this is an observed risk only and the reasons why this occurs are still uncertain. Whilst BAME students in our study and their families contracted COVID-19, it is misleading to suggest that all BAME families live in deprived conditions or overcrowded housing. However, it has been shown that BAME populations have a higher poverty rate and lower educational attainments compared to White British populations. They often make less healthy lifestyle choices impacting nutrition and mental health. For example, only 2% of British households are crowded as opposed to 30% of Bangladeshi households (40). Multigenerational households with dependent children and extended families can limit access to utilities such as water and sanitation and close proximity of living serves to promote transmission of the disease as social distancing measures cannot be observed (40). Whilst this might be an issue for some BAME students in this study, one cannot generalise as other BAME parents who contracted the virus did not transmit it to their families. So, it would be inappropriate to make assumptions and state that it is due to their living conditions only or their socioeconomic status.

### Maintaining effective infection control practices

As the pandemic was declared, many public health messages were conveyed to the general public. A few studies have been published on students’ knowledge, perceptions, and attitudes regarding COVID-19. A number of studies on student populations in various countries have shown that the majority had an awareness of COVID-19, often through social media, but not all were aware of appropriate preventative measures (44–48). Those with scientific backgrounds tended to be better informed, particularly females (45–50). This was evident in our study where White females who were not studying health-related subjects were unaware of how the virus was transmitted as opposed to all BAME females who studied health care related subjects. It has been suggested that those from higher socioeconomic status are better informed (51–54). However, there was no evidence to support this in this study group where 8 of 12 BAME participants received bursaries and were from low socioeconomic backgrounds. The reason may be due to the fact that these females were all reading professional healthcare degrees and hence were better informed about the epidemiology of this virus (50).

In terms of practising effective infection control, in the UK, White male students were found to be more likely to not wear a facemask and/or avoid social distancing in public places (55). These findings differ from this study where the majority of students from all backgrounds and genders had enough knowledge about COVID-19 and took personal protective measures. White male students who were reading professional healthcare degrees had better awareness of infection control practices in comparison to those who were reading non-healthcare degrees (55). Handwashing was one of the preventative measures advised by governmental health agencies. Handwashing became more frequent amongst female students and some BAME females became obsessive about it in our study. The reasons for this are unclear but may be associated with their mental health status and/or their fear of passing the virus on to their family. Additionally, handwashing may have also increased because students in the health professions are aware that handwashing is effective in decreasing the spread of viruses (50). In addition, such behaviours can be driven by extensive media coverage as was shown during the H1N1 pandemic (56) since the students spent a great deal of time on social media watching news amongst other things.

### Impact of news about the virus on news and social media

The app on which students spent the most time on was TikTok© followed by Instagram© and Snapchat©. Some also joined LinkedIn © to search for job opportunities. BAME students spent more time on social media in comparison to White students with BAME females spending up to up to 10–12 h in some cases, mostly during the night. They kept contact with their circle of friends on a daily basis for support and social interaction. A study in China (32) examining students’ lives at home during the pandemic reported that most spent their spare time watching TV or playing computer games and just under a half actively looked for news on the pandemic. Students in another study spent a long time playing video games and subsequently, the researchers found their mental health state to worsen (57).

Having a social life and friends is a vital element of their life at university and this part of their life was threatened by the pandemic. So, it is not surprising to find that many spent excessive amounts of time on social media. A study amongst Bangladeshi students showed that females tended to suffer highly from anxiety and fear of the pandemic, with many also reporting sleeping problems, nightmares, and lack of support from friends (58).

In our study, the majority if not all students expressed the sadness and anxiety they felt about the daily death rates on the news, and many stopped watching as they considered it was affecting their mental health. Most trusted BBC news broadcasts because it was evidence-based and expressed concerns and anger at the infodem (59, 60) and fake news circulating on social media at the time.

Overall, there were no differences between genders or ethnic groups in how students slept and its impact on their mental wellbeing. Common factors they all share included long hours spent on social media platforms, reading “fake news” which many students claimed made them anxious, and a more sedentary lifestyle, all of which are associated with anxiety and depression (61). In terms of relationships with their families and friends, most students indicated that they relied on friends for mental wellbeing support and were appreciative of financial support from their families. Their socioeconomic status and the physical environment and its impact on their wellbeing could also be a factor in how they experienced the COVID-19 pandemic lockdown.

### Xenophobia and racism

The spread of COVID-19 resulted in hostile sentiment globally toward Asian people (62–65). In Hong Kong, the Mandarin speaking people were not served in some restaurants (62, 63). Mandarin is mainland China’s official language whilst it is Cantonese in Hong Kong. An article in The New York Times reported anti-Chinese sentiment and xenophobic behaviours in many countries (62). One of the students in our study was of Chinese ethnicity and was subjected to racial abuse. He was left feeling anxious and frightened to travel on public transport. He was later diagnosed with PTSD as a result of his experience. He still feels extremely anxious when travelling on public transport and actively avoids groups of White youths. It is unfortunate that a state dignitary who referred to COVID-19 as “Chinese flu” led to an increase in xenophobic behaviour (63). Female BAME students in this study were also concerned about how the Government’s regulations on “no family get togethers” for Eid and Diwali celebrations and differential recognition of cultural festivals due to rises in COVID-19 cases in BAME communities also fuelled anti-Asian sentiment as Asians were blamed for spreading the virus (66, 67). A leading government figure praised Muslims for following social distancing rules during Ramadhan and then for political reasons imposed a lockdown on the celebration of Eid for Britain’s Muslims 3 h before it was due to start (66, 67). BAME females in this study viewed this as unjust, especially as Christmas was celebrated and people were allowed to see their families even though only for 1 day. Infact, some anti-Asian feeling was expressed in some British newspapers (66–69).

Some students also expressed concerns regarding the deaths of BAME doctors and nurses in the NHS. In fact, the first 11 COVID-19-related deaths of doctors in the UK were from BAME backgrounds (70). Around 21% of the NHS workforce is BAME and around 63% of the deaths due to COVID-19 were of BAME ethnicity (71–74). A report published in the Nursing Times described how BAME nurses were specifically targeted and forced to take shifts on COVID-19 ward, (75) highlighting cases of institutional racism. The PHE report “Beyond the data: understanding the impact of COVID-19 on BAME groups” implicates racism and discrimination due to marginalisation, as causal factors of increased risk of exposure to and death from COVID-19 (76). A report by the BMA suggested that systemic discrimination, bullying, and harassment of BAME staff in the NHS prevented them from speaking out about the lack of PPE potentially leading to an unequal COVID-19 burden amongst BAME people (77), One BAME student in our study was working in an NHS pharmacy and stated that there was a shortage of PPE and they could not get hold of more masks thus putting them at risk of catching the virus.

### Vaccinations

Since the outbreak of the pandemic, and after this study was undertaken, several vaccines for COVID-19 have been developed (78). According to a mathematical modelling study, a total of 19.8 million excess deaths (as an estimate of the true extent of the pandemic) are believed to have been averted between 2020 and 2021 across 185 countries worldwide; this represents 63% global reduction during the first year of COVID-19 vaccination (79). In the UK alone, the public health campaign of getting everyone vaccinated against COVID-19 has resulted in averting over 82,000 hospitalisations up to August 2021 and prevented between 102,500 and 109,500 deaths up to late August 2021 (80).

A global review of 19 studies conducted on approximately 75,000 healthcare professionals found an average of 22.5% expressed concerns about receiving COVID-19 vaccinations (81). Although a majority of the students in our study were happy to be vaccinated when interviewed (although the vaccines were not available to everyone at this time, only the most vulnerable received the first doses of the vaccine which was initiated in the UK from 8 December 2020) (82), a few from both ethnic groups and genders were hesitant to do so. However, some of the BAME students stated that their parents preferred to rely on traditional remedies which they believe to be effective in some African and South Asian countries (83, 84), whilst students of higher socioeconomic groups and White ethnicity were all in the favour of having vaccinations.

### Educational dimension

As a result of the lockdown, HEIs moved to online learning, teaching, and assessments. It has been estimated that this impacted 94% of the global student population (1.5 billion learners) (85). Academic staff faced the challenge of not just delivering lectures online but making learning active, engaging, and inclusive. Aristovnik et al. (17) carried out an online survey of over 30,000 students from 62 countries, excluding the UK. It was found that the majority were satisfied with the online mode of learning delivered as real-time video conferences, video recordings, and written communication. In contrast, in our study, students complained that some pre-recorded lectures had not been updated from previous years; the sound quality was poor and was not uploaded in a timely manner allowing students an opportunity to review the content prior to the lecture. They also expressed concerns about the increase in workload they faced. This was also found in approximately half of the sample population from Europe and Oceania, but not those from Asia and Africa. Concerns with video conferencing likely result from a lack of infrastructure in the countries affected (86).

Learning from home requires a suitable environment and adequate access to a computer and the internet and a great deal of discipline and motivation on the student’s part. Although all students had access to the internet, it was a general finding that BAME students did not have a quiet learning area making it difficult to maintain interest in lectures and to participate in them in our study. It should be noted that one BAME female could not afford her own laptop and was using her mobile phone to access online learning material. Her HEI had not clearly communicated to their students that laptops would be made available to students should they need them, and it wasn’t until this was pointed out to her by her academic advisor, that she had access to one. This should have been communicated early on to students in such circumstances. These findings are in agreement with studies in other parts of the world, where a lack of funding and resources to enable adequate delivery of online learning was a key factor in how students accessed and learned online material (17, 86–88).

In agreement with other studies (17, 86–88), students in our study complained that the etiquette of online learning, such as no talking in the background, switching off microphones whilst not in use, raising hands to ask questions, and general consideration of others, was not followed. In addition, some commented that little time was allocated for questions and that lecturers were sometimes reluctant to engage during the online lectures. Only a few students were therefore satisfied with the level of support they received which contrasts markedly with the findings of Aristovnik et al. (17). Although the switch to online examinations with 24 h to complete the assessment was universally welcomed by participants in our study, many commented on the lack of information they received from their HEI about the structure and requirements of these assessments.

A few students in our study also complained about having placements and experiential learning online as they felt that no real thought had been given to how sitting in front of a screen would ever replace face to face contact with patients or how geographical and time zone differences would be taken into account for international students, who found themselves involved in teaching sessions from midday to well past midnight. These findings are not dissimilar to a study on Japanese medical students which reported that anxiety and depression were more likely to manifest in students who had concerns about the transition to online learning and who felt that this form of education was not as effective as face-to-face lectures (17, 57). However, some also indicated that their learning improved due to online learning and their exam grades had improved. This may be partially attributed to the fact that they had online and open book exams. This is a different experience as was mentioned by many students compared to sitting in an exam hall and closed book exams with the latter as quoted as being more “stressful” by some students in our study.

In 2019 and 2020, there were just under half a million students in UK HEIs with a learning need of some description (89, 90). About 25% of students in this study had some form of learning disability (89, 90). A minority of these from both genders and ethnicities mentioned a lack of online support for dyslexia, for example. Added to this, their host HEIs were very slow in responding to requests for support. As pointed out by Kupper et al., individuals with disabilities of any nature tend to feel even more isolated in situations such as a pandemic (90). At the time of this study, there existed no evidence of HEIs planning for a pandemic situation which needs to be a part of their policies. Since then, several recommendations have been published by various organisations (91–93).

### Education and student identity

The higher education environment is the place where interaction with peers, staff, and the curriculum enables a student to construct a “student identity” which develops as a result of a sense of belonging to a learning community (94, 95). To date, no studies have been carried out to assess the effect of the COVID-19 pandemic on student identity so our study focuses briefly on what defined the identities of my survey cohort and how this changed during the pandemic.

Students in our study had already formed “a construct” of their student identity as none of them were freshers (94). The “construct” of identity is based upon various factors such as the environment and achievements of academic learning, which if positive helps construct a positive identity. It is also in part about interactions between them, their peers, and the staff; the curriculum is engaging, interesting, and intellectually stimulating and provides opportunities for training in specialist topics. Student identity further develops as a result of a sense of belonging to a learning community (94, 95).

As a result of the pandemic and isolation, students were now experiencing a loss of this identity has moved back into their family homes and losing daily contact with their peers. When asked if they felt they had lost their student identity as a result of the COVID-19 pandemic, the majority acknowledged that they had. It was interesting to note the reasons for this. The majority of BAME females associated their student identity with meeting friends and studying in the library. For White females, their identity was more related to being members of societies and socialising, being part of a community was clearly important to them. For these participants, student identity was clearly equated with a social life, at least initially, and was not academically focused. White males were more pragmatic in their views as they felt they had done the “clubbing” scene in their first or second year at university and were content to be where they were currently. BAME males were more worried about academic achievement and their future prospects.

### Effects of COVID-19 on mental health of students

Isolation and quarantining can lead to mental distress and more commonly anxiety and depression but is a necessary public health measure to prevent the transmission of infectious diseases (96). Several studies have reported the adverse effects of COVID-19 on people’s mental health during the lockdown as a result of being forced to live in an environment with restricted freedom of movement (96–99). Mental distress can impact all aspects of an individual’s life and for students, this can affect their motivation to study and socialise with their peers. In some, it can also influence their choices and behaviours, often to their detriment (98).

The effects of COVID-19 on the mental health of students in various countries around the world, but not the UK, have been summarised in a recent systematic review (97). The headline findings were that many students suffered mild to severe levels of stress and anxiety, some were recognised to be suffering severe depression and females were more likely to be affected than males. The main causes of these issues were identified to be worry about their financial status, educational attainment, future prospects, and living with family. Very similar findings were reported by Evans et al. (98) who surveyed students in the UK. Over half of the participants felt that their mental health had been affected by the pandemic and a third were classified as being clinically depressed. The majority were worried about being infected with the virus and passing it on to their families, something that was also evident in the findings of this study but felt that they had adapted well to social distancing and isolation. Some students in this study admitted that they found it difficult to always observe the rules on social distancing, but all ensured they wore facemasks when in public spaces.

The study by Evans et al. (98) did not examine whether there were differences in the prevalence of mental health problems between ethnic groups. Our findings show that females and BAME females in particular are affected more in this regard than their White counterparts. This seems to be associated with being from lower socioeconomic backgrounds, living in larger family groups, losing the autonomy they gained on entering higher education, and not having sufficient personal space to study and relax. Having said that, it should be noted that the majority of BAME females in this group had been diagnosed with mental health problems prior to the onset of the pandemic and were already receiving pharmacotherapy and/or counselling. Although their conditions were enhanced during the pandemic this raises the question of whether mental health issues were more prevalent amongst BAME females in the population at large prior to the pandemic. This might be the case given the findings of a longitudinal survey of measures of mental health in UK households (99, 100). It was found that females, both White and BAME, and BAME males experienced higher levels of mental distress than White males. Clearly, this is an area that warrants further study.

### HEI management of COVID-19

Of the many recommendations published by the WHO on the management of COVID-19 and any future pandemics, the two that most applied to the HEIs included (1) COVID-19 infodemic management and (2) building trust through risk communication and community engagement (93). Whilst the HEIs had implemented infection control and prevention measures as per the UK government’s recommendations, many had lacked in the aforementioned areas. There is also very little direct information on supporting marginalised groups of students. Certainly, this has been observed in a recent publication that highlights the inequalities faced by diverse cohorts of students (101).

Organisations such as UK Universities (91) have approached students to identify how best to support them, but their reports lack any tailored support for marginalised populations of students. An area of recommendation for HEIs is to develop policies that are streamlined to better support such cohorts. One of the areas that HEIs should focus on is how they manage infodemic by liaising with reputable healthcare organisations to eliminate confusion and misinformation which was a huge source of perceived anxiety for many students. Whilst the “lessons from the pandemic” publication mentioned various areas for improvement, mainly focusing upon how students wanted to learn, they fail to address the access to mental health services or even suggest if and how they can provide outreach services to students under such circumstances (91–93). The provision of accessible mental health services for students including outreach services that provide adequate support in a timely manner (100) should be provided. The provision of culturally appropriate mental health support from diverse backgrounds that provides tailored support for BAME students should be provided, as was highlighted in another qualitative study conducted amongst ethnic minority adults aged 18 to 65 years old, but which was comprised of only four students out of 30 participants interviewed (101). Universities should develop curricula that are easily delivered *via* online platforms along with the provision of guidance on financial issues and support if required. Whilst many studies have addressed this issue as highlighted in the discussion, HEIs should be particularly mindful of students’ socioeconomic status and their means to afford technology in terms of personal laptops and associated internet costs (102–106). Additionally, publications since have shown that students have not been completely satisfied with their universities in the way in which information was communicated (as only 43% felt it was clear) and how online learning was carried out during the pandemic (with only 49% feeling very or quite satisfied). The provision of relevant, timely, and focused communication to the students, as well as learning disability support alongside what is expected of students should be clear and unambiguous, particularly for international students (103, 104).

The COVID-19 pandemic has highlighted many inequalities and inequities experienced by marginalised groups and this is a timely opportunity for all HEIs to work collaboratively with all various student populations to eliminate these disparities. Further studies, perhaps employing a mixed methods approach, should be carried out on a much larger UK student population from many more universities to substantiate our findings and to inform policy-making and welfare provision for students.

## Conclusion

Students have suffered academically, socially, and financially as a result of the pandemic. This study set out to explore if there were any differences in the experiences of students from different ethnic groups and genders during the pandemic. Whilst all students were affected, BAME females were the most self-reported affected group in terms of suffering from mental health problems, probably because of living at home during the pandemic. Additionally, a lack of appropriate support from HEIs added to the burden of these students. HEIs, social services, student support, and government bodies should collaborate to improve policies relating to student welfare. In general terms, the experiences of BAME and White students were different in how they experienced life during the pandemic.

### Bias and reflexivity

An element of unintentional bias may or could have possibly been introduced by the researcher who did the questioning since she herself is a BAME female. However, the researcher had attended a previous workshop on interview techniques, and this was useful in raising her awareness of personal bias. A further potential bias relates to when the interviews were conducted. Students were reflecting upon their experiences from over a year or more ago as the COVID-19 pandemic lockdown began in March 2020 and continued until April 2021 and the interviews took place between November 2020 and May 2021. Hence there may have been some recall bias. The sampling methods used to recruit students may also have introduced bias as students were known to some colleagues in different universities, however, this was mitigated as the researcher did not know many of them. Finally, this research was not funded and therefore there was no bias has been done in its design and purpose.

### Strengths and limitations

Several limitations were identified in this study. One limitation included interviewing participants on a video conferencing platform since it does not allow a personal connection with the participant and clues to their feelings evident from their body language may also be missed. Another limitation was perhaps having just one gender-fluid student and not being able to have more persons in this category, since their experiences may have been different, and this may subsequently have implications for the support they needed. Additional limitations include that a majority of the students who participated were pursuing professional degrees, -most of which were health-related. As such, not only were the students not representative of UK post-secondary students, but they also would be better informed about COVID-19 and more likely to follow advice on how to decrease the possibility of transferring the virus which may have affected the reliability of some of the data. For example, this may explain why there was an increase in hand washing, in contrast to being the result of a mental health issue. Furthermore, the limitations have to mention that the sample was small and that the qualitative research was conducted before there were vaccines available for COVID-19 and that both things may have affected the reproducibility of the results. However, a strength of this study is that it is one of the very few studies that explore students’ experiences and compares these between two ethnic groups enabling the identification of differences in the experiences between different ethnic groups.

## Data availability statement

The raw data supporting the conclusions of this article will be made available by the authors, without undue reservation.

## Ethics statement

The studies involving human participants were reviewed and approved by Brighton and Sussex Medical School Ethics Research committee. Informed recorded verbal consent was obtained from each participant due to the pandemic restrictions. Verbal consent for the publication of any potentially identifiable data was obtained from the participants in the form of audio-recordings due to the pandemic restrictions.

## Author contributions

GH collected and analysed the data which was verified by SZ. Both authors contributed to the manuscript, conceptualization of the study, and approved the submitted version.
