# Supplementary material for: COVID-19 pandemic experiences of students from BAME and White ethnic groups pursuing higher education in the UK: A qualitative comparative exploration
Source: Front Psychiatry. 2023 Feb 23;13:1078479. doi: 10.3389/fpsyt.2022.1078479 (PMC9996017; doi:10.3389/fpsyt.2022.1078479)
Supplement: Supplementary file 1 [file Data_Sheet_1.PDF]

## Appendices

## APPENDIX 1: SEMI STRUCTURE INTERVIEW PROTOCOL

The framework was adapted from Kallio et al., 2016.The following semi-structured interview guide was adapted from Zubin and Gregory, 2020 (Fig. 1).The following four themes were used:

1. Demographics
2. Pre COVID-19 life (social, economic and general wellbeing)
3. Life during lockdown (social, economic and general wellbeing)
4. Post COVID-19 expectations from life (social, economic and general wellbeing)

**Outline and Introduction of study protocol**

The researcher will provide a brief explanation of informed consent and data protection for interviewrecording and confidentiality, and explain that there is no requirement to answer all questions. Explain that checking of the interview transcript with the project supervisor is a requirement and sending this to participate. If choosing to participate, there is no requirement to answer all questions but checking of the interview transcript with the project supervisor is a requirement. The interview transcript will be sent to the participant for checking in the event of any data to be removed at the individual's request.

**The following questions were addressed during the interview:**
**Demographics**: age, sex, ethnicity, year of HE studies, residential location (geographical area in theUK).

**Figure 1. Semi structured interview protocol**

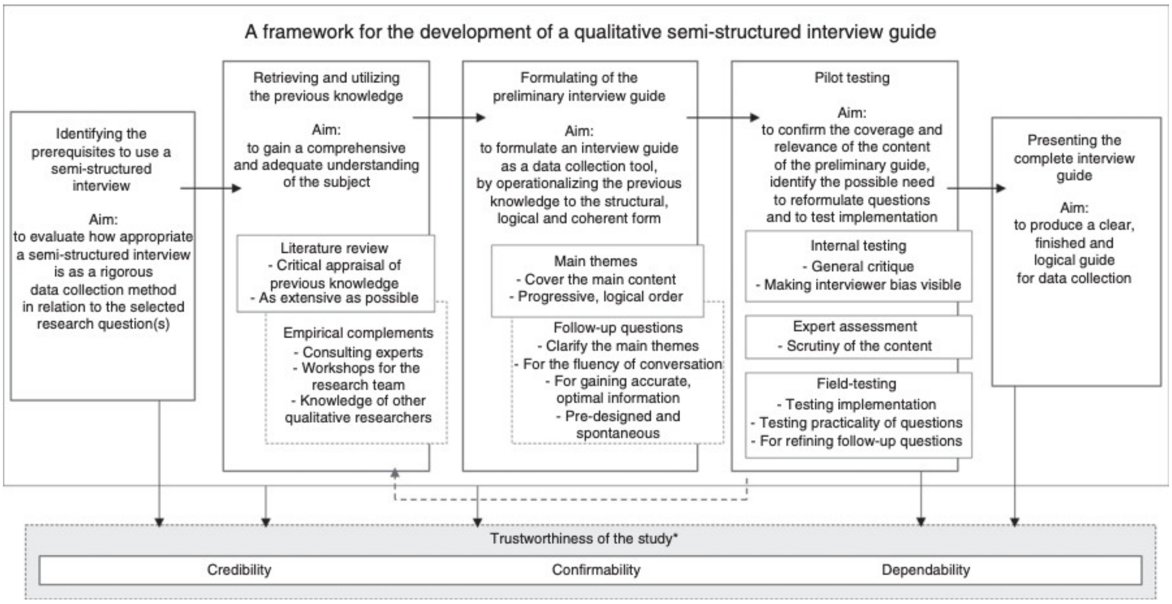


**Pre-COVID-19 life**
I started by asking the participant to reflect upon a typical day in their life pre COVID-19 lockdown using aides-mémoires: HE life - lectures, socialisation, nature of socialisation with friends /family, employment status, personal

relationships, recreation time/activities.
Were you living at home or on campus or in rented accommodation? Were you working to support yourself?Where were/are you working? What was your personal hygiene routine before the pandemic started?

**Perceptions of the COVID-19 virus**
Did you ever think we could be in a pandemic situation?Had you ever heard of this virus before lockdown? How do you think it is transmitted? Do you think enough precautions are being taken to prevent infection? Do you think you can catch the virus from surfaces such as books and desks?How do you personally protect yourself from this virus?
How did your HEI adapt? How did your own HE life change? How did your day-to-day university / social/ work life evolve during this time?
Did you feel enough help /support/ guidance provided by the HEI during this time? What helped you during this period? (prompt personal relationships, government guidelines, family, How did your HEI adapt? How did your own HE life change? How did your day-to-day university / social/ work life evolve during this time? Did you feel enough help /support/ guidance provided by the HEI during this time? What helped you during this period? (prompt personal relationships, government guidelines, family, HE advice /support, signposting to general wellbeing campaigns, your work /employment support)? How did this affect your personal hygiene routine during the pandemic? Can you reflect on why this was helpful?  What was done that was not helpful? Can you reflect on why this was not helpful? Reflecting on that first announcement, what could have or should have been done differently to provide support for students to deal with this situation? It has since become evident that this pandemic is here to stay for some time. What is your response to this? What did you expect to happen in the days, weeks, and months ahead? What actually has happened? How has this impacted upon your life an life in general? What helped you during this period? Can you reflect on why this was helpful? What was done that was not helpful? Can you reflect on why this was not helpful? How much time did you spend on social media during the pandemic? Describe your feelings to listening to the news on the media. On March 16, 2020, the government announced a state of lockdown due to COVID-19.  How did you initially respond to this announcement? What was the response amongst your friends /family? Were you living at home or on campus or in rented accommodation when this was announced? What were you expecting to happen in the days, weeks, and months ahead during the lockdown?
**HE advice /support, signposting to general wellbeing campaigns, your work /employment support)**
How did this affect your personal hygiene routine during the pandemic? Can you reflect on why this was helpful? What was done that was not helpful? Can you reflect on why this was not helpful?
Reflecting on that first announcement, what could have or should have been done differently toprovide support for students to deal with this situation?
It has since become evident that this pandemic is here to stay for some time.What is your response to this? What did you expect to happen in the days, weeks, and months ahead?What actually has happened? How has this impacted upon your life an life in general?What helped you during this period? Can you reflect on why this was helpful? What was done that was not helpful? Can you reflect on why this was not helpful?
How much time did you spend on social media during the pandemic?Describe your feelings to listening to the news on the media. On March 16, 2020, the government

announced a state of lockdown due to COVID-19.How did you initially respond to this announcement? What was the response amongst your friends /family? Were you living at home or on campus or in rented accommodation when this was announced? What were you expecting to happen in the days, weeks, and months ahead during the lockdown?

**Life during COVID-19**
How did your HEI adapt? How did your own HE life change? How did your day-to-day university / social/ work life evolve during this time? Did you feel enough help /support/ guidance provided by the HEI during this time? What helped you during this period? (prompt personal relationships, government guidelines, family, HE advice /support, signposting to general wellbeing campaigns, your work /employment support)? How did this affect your personal hygiene routine during the pandemic? Can you reflect on why this was helpful? What was done that was not helpful? Can you reflect on why this was not helpful? Reflecting on that first announcement, what could have or should have been done differently toprovide support for students to deal with this situation? It has since become evident that this pandemic is here to stay for some time.What is your response to this? What did you expect to happen in the days, weeks, and months ahead? What actually has happened? How has this impacted upon your life an life in general? What helped you during this period? Can you reflect on why this was helpful?What was done that was not helpful? Can you reflect on why this was not helpful? How much time did you spend on social media during the pandemic?Describe your feelings to listening to the news on the media. On March 16, 2020, the government announced a state of lockdown due to COVID-19.How did you initially respond to this announcement? What was the response amongst your friends /family? Were you living at home or on campus or in rented accommodation when this was announced? What were you expecting to happen in the days, weeks, and months ahead during the lockdown?

I will then ask the participant to reflect back upon when their HEI announced a lockdown, beginning in March 2020.
**Many HE institutions have elected to remain open during the pandemic.**
How is your HE institution /workplace different today, compared with pre COVID-19 lockdown?How safe do you feel coming to the campus? Do you feel adequate precautions are in place to prevent transmission of this virus?How do you think the virus is transmitted? What changes do you see as positive and potentially worth preserving in the future?What changes have been difficult and should be abandoned as soon as possible? What advice would you give to your friends, family, partners, HE institution and government leadersand employers about how best to manage situations like this in the future, particularly as we are in the middle of experiencing a second wave of COVID-19 infections? Do you think there was too much information on the media about the pandemic?How does this make you feel?

How have you coped or managed your personal life during these last few weeks? What has helped you? What has not been helpful? What would you do differently, knowing what you know now?
Personal life – optional questions (participants are not required to answer this section)

**Final wrap -up question**

Is there anything else you would like to share with me regarding your personal student/employee/family experience of COVID-19?
Thank you for participating in this study. A transcript of this interview will be produced and sent toyou for review. If after reviewing it, you would like to provide any other information/details/clarification, please let me know.

brighton and sussex medical school

## APPENDIX 2: PARTICIPANT INFORMATION SHEET

**A Qualitative Study of the Impact of COVID-19 in BAME and White British students inHigher Education in the UK**

**Dear Participant**

'*You are being invited to take part in a research study. Before you decide whether or not to take part, it is important for you to understand why the research is being done and what it will involve.Please take time to read the following information carefully*'.

## WHAT IS THE PURPOSE OF THE STUDY?

This study aims to explore the views of students from two ethnic groups (BAME and White British) of the impact of COVID-19 on their daily lives. The new coronavirus “COVID-19” from Wuhan, China in late 2019 is now the cause of one of most disruptive pandemics worldwide. In early March 2020, the WHO declared a global pandemic. To date, this pandemic has caused 39,596,858 confirmed cases of COVID-19, including 1,107,374 deaths.

UK has imposed travel restrictions along with closure of many businesses, and venues such as gymnasiums, theatres, cinemas, bars and restaurants in order to contain the transmission of COVID-19. As there were signs of the numbers of infections going down, after an initial lockdown period, many businesses and venues were allowed to open up for business. Universities across the UK opened,having being provided guidance from the government in readiness for the new academic year with many students returning to life on campus.

The pandemic affected everyone and as such one affected group is 18-25year old students in higher education as has been shown in few publications globally. Studies globally on university students have shown that the students suffered from psychological stresses resulting in mental health illnessessuch depression and anxiety. Many lower-income students expressed concerns over graduating, employment prospects and financial problems. The COVID-19 pandemic has impacted highly upon the socioeconomic factors in this student population leading many lower income students to either withdraw from the classes or change their degree programme. Not much research on impact upon students has been published in the UK, even though a lot of research has been published around the impact of COVID-19 on vulnerable groups, people with co-morbidities or frontline healthcare workers, both in the UK and globally, but there is a dearth of literature on the impact of COVID-19 on students and especially on those from the UK’s BAME and White British ethnicity backgrounds. The UK’s King’s Fund which is a charitable organisation, response to the Public Health England report, “Disparities in the risk and outcomes of COVID-19” [18] in particular states, that *“The coronavirus pandemic has exposed the stark inequalities that exist throughout our society. People who have been worst affected by the virus are generally those who had worse health outcomes beforethe pandemic, including people working in lower-paid professions, those from ethnic minority backgrounds and people living in poorer areas. We’ve known for many years thatthese groups typically have worse health*

*outcomes, but there has been disappointingly little effort overthe past decade to address inequalities and improve people's health. The scandal is not that the virus has disproportionately affected certain groups, but that it has taken a global pandemic to shine a light on deeply entrenched health inequalities".*

Thus, taking into account the difference in socioeconomic factors that may impact upon BAME students in the higher education sector UK, my aim is to highlight the impact of COVID-19 on BAME students, inparticular, the impact on their social, economic and general wellbeing and compare this with impact on WhiteBritish students.

**Research question:**
The research question is "Has COVID-19 pandemic had a greater impact on the socioeconomic and general wellbeing status of students from BAME ethnic backgrounds compared to White British students?

**WHY HAVE I BEEN INVITED TO PARTICIPATE?**
You have been invited to participate because you fit into either one of the 2 specified ethnicgroups and study in a higher education institution in the UK.

**DO I HAVE TO TAKE PART?**
*'It is up to you to decide whether or not to take part. If you do decide to take part you will be giventhis information sheet to keep and be asked to sign a consent form. If you decide to take part you are still free to withdraw* ***at any time leading up to the day/moment*** *of the interview taking place and without giving a reason'.* **However, once your data is in publication, it will not be possible to withdraw from the study.

By choosing to either take part or not take part in the study there will be no impact on yourmarks, assessments or future studies.

**WHAT WILL HAPPEN TO ME IF I TAKE PART?**
You will be asked to participate in a one to one in depth interview which will last up to 45 minutes and engage in a conversation on your previous (pre pandemic) and current experiences (during pandemic) and expectations of life (post pandemic) as a university student. The interview will be recorded with your consent and confidentiality will be maintained since all data will be encrypted inrequirements with the GDPR regulations and the Data Protection Act. Your identity will be anonymised by encoding your name as W1or B1.

**WHAT ARE THE POSSIBLE DISADVANTAGES AND RISKS OF TAKING PART?(WHERE APPROPRIATE)**
There are no risks involved as such, but it may take up to 50 minutes of your time for the interviewto be conducted and another hour or so when the transcript of the recording will be sent to you forverification. There are no financial or any other incentives for participants.

**WHAT ARE THE POSSIBLE BENEFITS OF TAKING PART?**
The benefits include contributing towards a study where only anecdotal evidence is quoted on students' experiences in the UK and no such published studies have emerged. The findings may lead to development or informing policies or guidance for organisations on supporting students during such times.

**WILL MY INFORMATION IN THIS STUDY BE KEPT CONFIDENTIAL?**[1]

All participants will be anonymised and confidentiality will be maintained at all times by giving eachone a code ie B1 or W1. All data will be kept in an encrypted recording only made accessible to meand the supervisor on request. Any written information will be encrypted and stored on an passwordprotected computer. All data will be deleted on completion of the research project once it is published.

[1] As a researcher, **two** principles are important here. Firstly the '*Common law duty of confidentiality*' by which all participants, unless specifically informed otherwise can expect that theresearcher will treat what they have received with confidentiality. Secondly, the requirements of *UK and EU data legislation* that apply to all types of personal data that are received. Failure to actin accordance with the principles and requirements of either may be considered research misconduct.

brighton and sussex medical school

## APPENDIX 3: CHECKLIST CONSENT FORM FOR PROJECT PARTCIPANTS

**Title of Project: A Qualitative Study of the Impact of COVID-19 in BAME and White Britishstudents in Higher Education in the UK**

Name of Researcher and School: Removed
**RGEC Ref no: V1**

| | ***Please tick box*** | |
|---|---|---|
| | **YES** | **NO** |
| I consent to being interviewed by the researcher | | |
| I agree to allowing the interview to be filmed / audio-recorded | | |
| I understand that I will be given a transcript of data concerning me for my approval before being included in the write up of the research | | |
| I understand that any information I provide is confidential, and that no information that I disclose will lead to the identification of any individual in the reports on the project, either by the researcher or by any other party | | |
| I have read the information sheet, had the opportunity to ask questions and I understand the principles, procedures and possible risks involved. | | |
| I consent to the processing of my personal information and data for the purposes of this research study. I understand that such information will be treated as strictly confidential and handled in accordance with the General Data Protection Regulation (GDPR) 2016. | | |
| I understand that my participation is voluntary, that I can choose not to participate in part or all of the project, and that I can withdraw at any stage of the project without being penalised or disadvantaged in any way | | |
| I agree to take part in the above research project | | |

Name:

Signature:

Date:

brighton and sussex medical school

## APPENDIX 4: CHECKLIST VERBAL CONSENT FOR RESEARCH PARTCIPANTS

**Title: A Qualitative Study of the Impact of COVID-19 in BAME and White British students inHigher Education in the UK**

Name of Researcher and School: Removed

**RGEC Ref no: V1**

A. **Process of obtaining verbal consent from research participants**
B. Give an account of how you will verbally **explain** to the research participants as clearly aspossible and in terms that they are familiar with:

- The **aims** and **objectives** of your research;
- The reasons why you have **selected** them for this research;
- The reasons why their story/knowledge/understanding/opinions are **relevant** to your research;
- The ways in which the research data will be **used**: for example in a dissertation/publication/blogs/thesis

Dear Participant

My name is XXXXX and I am a student at BSMS studying for an MSc in Global Health. Iam required to conduct a research project as part of my fulfilment criteria to obtain the MSc.As such I have decided to investigate the impact of COVID-19 in BAME and White British students in higher education in the UK.

**Aim of my study:**

My research question is "Has COVID-19 pandemic had a greater impact on the socioeconomicand general wellbeing status of students from (Black, Asian, Ethnic Minorities) BAME ethnicbackgrounds compared to White British students?"

**Reasons:**

The recent Public Health England report, "Disparities in the risk and outcomes of COVID-19"has been not very transparent in its entirety causing a renowned independent charitable organisation working to improve health and care in England, called The UK's King's Fund response to the Public Health England report, "Disparities in the risk and outcomes of COVID-19" in particular states that *"The coronavirus pandemic has exposed the stark inequalities thatexist throughout our society. People who have been worst affected by the virus are generally those who had worse health outcomes before the pandemic, including people working in lower-paid professions, those from ethnic minority backgrounds and people living in poorer areas. We've known for many years that these groups typically have worse health outcomes, but therehas been disappointingly little effort over the past decade to address inequalities and improve people's health. The scandal is not that the virus has disproportionately affected certain groups, but that it has taken a global pandemic to shine a light on deeply entrenched health inequalities*"

Additionally, a few studies conducted globally have shown that the impact of COVID-19 On University students has shown students to be highly concerned about their financial loss as they have concerns about graduation and future employment prospects. Additionally, manyhave been unable to continue with their current education due to job

losses and unable to provide financial support for their education. As a university participant, your experiencesof this pandemic will be vital towards providing an insight in one of the first such studies carried out in the UK and its potential impact upon provision of more guided support for this population group as a result. It will also highlight any variability in terms of socioeconomicimpact upon both ethnic groups. This qualitative study will be carried out by conducting one to one in depth interviews, conductedonline via Microsoft teams application or a similar online application and data will be used to inform further research with a view to informing future policies that support student welfare and completion of their undergraduate studies.

1) I will **verbally explain** to the research participants:
   a).That they can **withdraw** from the research at any time without giving a reason, and without being penalised or disadvantaged in any way and/or that they can tell me notto use certain types of information at any time;
   b).What **confidentiality** means in the context of the research and how confidentiality will be maintained in this particular context (OR explain why confidentiality cannot be maintained in this particular case – e.g. focus group);
   c). What **anonymity** means and how it will be maintained in this particular context (OR Iwill ask approval for the use of their name/location/company/organisation in the final report/dissertation/further publication).
2) Describe below how you will **verbally explain** to your informants that they can **withdraw**from the research at any time, what **confidentiality** and **anonymity** mean in your researchcontext, and how you will explain these terms to your informants:
3) I will verbally **check** with the research participants that:
   They are happy to be interviewed and/or observed by me;
   They are happy for me to be present at and/or participate in their activities;
   They are happy for me to take notes on the interview/observations/interactions;
   They are happy for theinterview/observation/interaction to be:
   1.photographed
   2.video-taped
   3.audio taped

   They are happy to be contacted again for a further interview should that be required.

   Where photographs, video or audio tape are to be shown to others, <u>specific</u> and separate consent should be sought in written, video or audio form. This consent should involve a full explanation of the kinds of contexts in which these media are to be shown

   Media should not be published – online or elsewhere – without specific consent.

4) I will give the research participants the opportunity to **ask any questions** about any of the above or any other concerns they may have.

5) I understand that seeking verbal consent will involve explaining all I have documented above and that verbal consent is an ongoing process. I understand that I will need to document the ongoing process of verbal consent.

## C. Researcher Training and Consultation of Professional Guidelines

1) I confirm that in the course of my research design, I have received the following training inthe ethical aspects of my research (please give details of any modules, CPD or other research ethics training you have attended in the last 18 months):

2) I confirm that I have read and carefully considered the ethical guidelines of the main professional associations for my subject area (eg: the ASA ethical guidelines: https://www.theasa.org/ethics/guidelines.shtml) and incorporated them into my research design.

Researcher

Signature:

XXXXX

Date:22/10/20

## APPENDIX 5: GATEKEEPER'S PERMISSION LETTER

**Title: A Qualitative Study of the Impact of COVID-19 in BAME and White British students inHigher Education in the UK**
**Dear Gatekeeper**

**I would like to invite you to assist me in conducting a research study. Before you decide you need to understand why the research is being done and what it would involve for youand for the participants. Please take time to read the following information carefully. Askquestions if anything you read is not clear or if you would like more information. Take time to decide whether or not to facilitate this research.**

**WHO I AM AND WHAT THIS STUDY IS ABOUT**

My name is XXXX and I am currently studying for an MSc in Global health at BSMS. As partof requirements to fulfil this degree, I am conducting a study which aims to explore the views of students from two ethnic groups (BAME and White British) of the impact of COVID-19 on their daily lives.

Here is some information on the study:

The new coronavirus "COVID-19" from Wuhan, China in late 2019 is now the cause of one of mostdisruptive pandemics worldwide. In early March 2020, the WHO declared a global pandemic. To date, this pandemic has caused 39,596,858 confirmed cases of COVID-19, including 1,107,374 deaths.

UK has imposed travel restrictions along with closure of many businesses, and venues such as gymnasiums, theatres, cinemas, bars and restaurants in order to contain the transmission of COVID-19. As there were signs of the numbers of infections going down, after an initial lockdown period, many businesses and venues were allowed to open up for business. Universities across the UK opened, having being provided guidance from the government in readiness for the new academic yearwith many students returning to life on campus.

The pandemic affected everyone and as such one affected group is 18-25year old students in higher education as has been shown in few publications globally. Studies globally on university students have shown that the students suffered from psychological stresses resulting in mental health illnessessuch depression and anxiety. Many lower-income students expressed concerns over graduating, employment prospects and financial problems. The COVID-19 pandemic has impacted highly upon the socioeconomic factors in this student population leading many lower income students to either withdraw from the classes or change their degree programme. Not much research on impact upon students has been published in the UK, even though a lot of research has been published around the impact of COVID-19 on vulnerable groups, people with co-morbidities or frontline healthcare workers, both in the UK and globally, but there is a dearth of literature on the impact of COVID-19 on students and especially on those from the UK's BAME and White British ethnicity backgrounds.The UK's King's Fund which is a charitable organisation, response to the Public Health England report, "Disparities in the risk and outcomes of COVID-19" in particular states, that *"The coronaviruspandemic has exposed the stark inequalities that exist throughout our society. People who have beenworst affected by the virus are generally those who had*

*worse health outcomes before the pandemic,including people working in lower-paid professions, those from ethnic minority backgrounds and people living in poorer areas. We've known for many years that these groups typically have worse health outcomes, but there has been disappointingly little effort over the past decade to address inequalities and improve people's health. The scandal is not that the virus has disproportionately affected certain groups, but that it has taken a global pandemic to shine a light on deeply entrenchedhealth inequalities*". Thus, taking into account the difference in socioeconomic factors that may impact upon BAME students in the higher education sector UK, my aim is to highlight the impact ofCOVID-19 on BAME students, in particular, the impact on their social, economic and general wellbeing and compare this with impact on White British students.

Research question:
The research question is "Has COVID-19 pandemic had a greater impact on the socioeconomic andgeneral wellbeing status of students from BAME ethnic backgrounds compared to White British students?

**WHAT I NEED YOUR ASSISTANCE WITH**
**I would like to request your assistance in the dissemination of the information regarding this study via email in the hope of recruiting students from your department. I need 12 students (from each one of the BAME and White British background respectively) depending upon whether they meet the inclusion/exclusion criteria as depicted here:**

Inclusion criteria
1. Students aged 18-25 years old and a student in a Higher Education institution
2. Participants should have White British (English, Welsh, Scottish, Northern Irish or British, Irish, Gypsy or Irish Traveller)
3. Participants should have BAME background (**Mixed or Multiple ethnic groups to include** White and Black Caribbean, White and Black African, White and Asian, any other Mixed or Multiple ethnic background; **Asian or Asian British to include** Indian, Pakistani,Bangladeshi, Chinese, any other Asian background; **Black, African, Caribbean or Black British** to include African, Caribbean, any other Black, African or Caribbean background
4. **Other ethnic group to include** Arab **or** any other ethnic group)
5. Participants should not be freshers
6. Fifty percent of all participants will be or have been previously employed either full or parttime.

Exclusion criteria
1. Anyone outside of the 18-25year age range and not in Higher Education
2. Participants who are "freshers" and not returning i.e. first year students
3. Participants from ethnic backgrounds other than those defined in the inclusion criteria

**Only myself and my supervisor will have access to the database which will be encrypted ascan be seen from the attached research protocol.**

**WHAT TAKING PART IN THE RESEARCH WILL INVOLVE?**
**I have attached the participants information sheet for you to see the finer details.**

**WHAT WILL HAPPEN TO THE RESULTS OF THE STUDY?**
**I hope to publish the results in a peer reviewed journal for dissemination to the wider public**

**WHO SHOULD YOU CONTACT FOR FURTHER INFORMATION?**
**Please contact my supervisorDr Xxxx**
**email address:**
**And myself XXX, email:**
**THANKYOU**

**Consent to facilitate research**

**□ I….................................................voluntarily agree to help facilitate this research study.**

**□ I understand that even if I agree to help now, I can withdraw at any timewithout any consequences of any kind.**

**□ I have had the purpose and nature of the study explained to me in writing and I havehad the opportunity to ask questions about the study.**

**□ I understand that I will assist in the dissemination of the information about the study viaemail to all students.**

**□ I understand that all data collected in this study is confidential and anonymous.**

**□ I understand that I am free to contact any of the people involved in the research to seekfurther clarification and information.**

**Signature of gate keeper**
**--------------------------------------- ----------------**
**Date**

**Signature of researcher**
**I believe the participant is giving informed consent to participate inthis study XXXX**
**Signature of researcher: xxxxx**
**Date:22/10/20**

## APPENDIX 6: CERTIFICATE OF ETHICS APPROVAL

brighton and sussex medical school

SREO BSMS SCHOOL

| Certificate of Approval | |
|---|---|
| **Reference Number** ER/GH216/4 | |
| **Title Of Project** A qualitative study of the impact of COVID-19 in BAME and White Britishstudents in higher education in the UK | |
| **Principal Investigator (PI): Student Collaborators**<br>**Date Of Approval Approval Expiry Date RGECChair**<br><br>**Name of Authorised Signatory Date** | Geeta Hitch<br><br>11-Nov-2020 10-Nov-2021<br>Prof Valerie Jenkins Ceri Butler 24-Nov-2020 |

The Brighton and Sussex Medical School Research Governance and Ethics Committee (RGEC) has assessed your application and granted Ethical and Research Governance Approval to proceed with the above named project.

**Approval is granted on the following basis:**
**Duration of Approval**
Approval covers the period stated above. Research must commence within 12 months of thecertificate start date; any delay beyond 12 months and this certificate of approval will lapse necessitating renewed review of the project.

**Project Amendments**
Any substantial changes or minor amendments to the project following issue of the certificate ofapproval should be submitted to the Research Governance and Ethics Committee for review andauthorisation prior to implementation. Please submit your application for an amendment to the Committee (via rgec@bsms.ac.uk) using the Request for an Amendment Form.

**Reporting Adverse and Unexpected Events**
Any incidents occurring during the project's lifespan presenting ethical and safety implications must be reported immediately to the Chair of the Research Governance and Ethics Committee. In the event of an adverse (undesirable an unintended) and unexpected event occurring during the project, research must be stopped immediately and events reported to the Chair of the Research Governanceand Ethics Committee within 24 hours of its occurrence.

**Monitoring**
The Medical School has a duty to ensure all its research is conducted in accordance with the University of Sussex's Code of Practice for Research and Research Governance and Ethical Review Framework. In order to ensure compliance auditing may be undertaken annually and /or periodic monitoring of a percentage of approved research studies. If your project is selected you will be given4 weeks' notice to prepare all study documentation for inspection.

**Notification of End of Study**
Please notify the Research Governance and Ethics Committee once the study has completed. It is also your responsibility to inform the Committee in the event of early termination of the project or ifthe work is not completed.

24/11/2020
